# Advance Care Planning Preferences in Hong Kong: A Cross-Sectional Study in a Community

**DOI:** 10.3390/healthcare10020384

**Published:** 2022-02-17

**Authors:** Andrew Yu

**Affiliations:** College of Arts, Humanities & Social Sciences, The University of Edinburgh, Edinburgh EH8 9JU, UK; andrew.ck.yu@ed.ac.uk

**Keywords:** advance care planning, aged, Hong Kong

## Abstract

(1) Background: Hong Kong is experiencing population aging, but there is little research on advance care planning. This research asks for community-dwelling older adults’ views on advance care planning in order to provide a different angle to the results collected from nursing homes and hospitals. (2) Methods: The targeted respondents were people aged 55 or above. A total of 282 questionnaires were collected using the random sampling method. Respondents were asked whether they would make an advance care plan, whether they would prepare for their death, and whether they had received any death education; they were also given eight statements related to treatments and decision making. (3) Results: The study found that only 17% of the respondents would like to use advance care planning, even after it was explained to them. Advance care planning favorers would be more likely to insist on having wills and would be more likely to trust doctors rather than family members. (4) Conclusions: The results also suggested that the medical care and social support for end-of-life patients should be improved. The results also showed the importance of medical professionals as they showed that respondents wanted advice on end-of-life decision-making from medical professionals.

## 1. Introduction

Everybody will die—death matters to everyone. Hong Kong is experiencing rapid population aging [1]. In 2016, 30% of the total population was aged 55 or above, and this figure is expected to reach over 40% by 2050 [2,3]. There has been active discussion in recent years about the concept of advance care planning. Advance care planning is a process that enables patients to express their preferences regarding end-of-life care, and it enables family members and healthcare workers to understand patients’ preferences and to make decisions on the behalf of patients when they are unable to do so [4].

Advance care planning preferences can be indicated in advance in the form of an advance directive [5]. An advance directive is a written statement that allows a person to indicate the form of health care he or she would like to receive in the future when he or she is no longer competent [6]. Many Western countries have already passed laws for AD. For example, the United Kingdom passed the Mental Capacity Act in 2005, which provides a legal framework for introducing advance decisions to refuse treatment [7]. In 1991, the United States passed the Patient Self-Determination Act, which requires all state-funded hospitals to have a policy on living wills and advise patients of their right to execute an advance directive [8].

Death is still a taboo subject that is difficult to openly discuss in Asian cultures. In traditional Chinese culture, mentioning death is sensitive and should be avoided [9,10]. Advance directives have not been legislated in Hong Kong presently. Advance care plans and advance directives were seldom discussed among healthcare professionals or the public in Hong Kong until 2006, when the Law Reform Commission recommended promoting the concept of advance directive through non-legislative means [11]. While there is no specific legislation for advance care plans or advance directives in Hong Kong, it does not mean that people cannot make an advance directive. Under the common law framework, people can establish their end-of-life care wishes in advance. A valid and applicable advance directive has legal status, and family members cannot override it [12].

Although there is a growing interest in advance care planning in Hong Kong, the studies of advance care planning in Hong Kong are limited. Many studies were conducted among elders in a nursing home or hospital setting by medical practitioners in Hong Kong [13,14,15]. For example, Chu et al. conducted a survey in 140 nursing homes in Hong Kong and found that despite the fact that many of them did not understand the idea of an advance care plan or advance directive, 88% of them would prefer to have an advance care plan or advance directive after they had been explained to them [13]. However, one of the major problems of conducting studies in a nursing home or hospital setting is that the respondents or the target samples will be ill or older people [14,15].

Chung et al. and Pang et al. have tried to conduct telephone surveys in Hong Kong [11,16]. Both studies found that respondents were mostly unaware of advance care planning and advance directives. Two studies also reported differences in the preference for using advance care planning. The study by Pang et al. found that 78% of people would use advance care planning after researchers explained the terms to them, and younger people and those with secondary education or above had a greater chance of adopting advance care planning [16]. The study by Chung et al. found that only 60% of people would use advance care planning after having it explained to them during the interviews, and they found that age and education level were not related to the preference for using advance care planning [11].

Telephone surveys, although less expensive than face-to-face surveys, have limitations, especially in Hong Kong. Time constraints are one of the disadvantages of telephone interview surveys. Some potential interviewees might be reluctant to participate in the survey because they fear it would take a long time. Therefore, the questions used in telephone surveys are generally short and simple. Furthermore, the quality of the collected answers is questionable because when researchers reach out to people at home, they are often engaging in other activities, such as eating dinner or watching television. There are also some unique issues to the telephone surveys in Hong Kong. For example, the sampling lists of telephone surveys are often fixed-line telephone numbers; however, in the 21st century, when mobile phones are popular, many households do not have a fixed-line telephone number. It is also not feasible to use mobile phone numbers as a sampling list—as mobile phone numbers in Hong Kong are not registered with real names, many people in Hong Kong have more than one mobile phone number, and the number of mobile phone numbers in use will not be proportionate to Hong Kong’s population.

While telephone surveys are convenient and inexpensive, the limitations of telephone surveys have become increasingly apparent as landline telephones have fallen out of fashion. Although the cost will be higher, community-based sample surveys are preferable to telephone surveys for many social researchers because the questions in community-based sample surveys can be more detailed and the investigators can be on-site to ensure that respondents answer the questions seriously. However, because discussing death is taboo in Chinese society, studying death in a community setting in Hong Kong is difficult. Such research is unlikely to be popular with communities, such as conservative town councilors or homeowners.

The concept of a ‘good death’ has emerged in Hong Kong over the past decade. Luk et al. have defined that a ‘good death’ means people have choice and control over where death occurs and who is present with them at the end [17]. Therefore, having the option of dying in place or in a familiar environment and of having the company of family members is important. In Japan, the government has promoted dying in place and terminal home care over the past decade, especially in rural areas [18,19,20,21,22,23]. However, in Hong Kong, death at home or dying in place is basically impossible due to high residential and population density. The majority of Hong Kong’s population die in hospital. According to Luk et al., this is because ‘people may fear depreciation of property value if the elderly die at home’ [17]. Furthermore, inappropriate admissions to hospital are common in Hong Kong. Almost all older patients with terminal diseases or irreversible chronic illnesses die in hospitals, as they are rarely referred to palliative care services [17]. As for the willingness to die at home, Chung et al. found that 68% of people would still be unwilling to die at home if they could get medical assistance at home. More than 90% are unwilling to die at home without medical assistance at home [11].

As most of Hong Kong’s residents will eventually die in hospital, people need to have a plan for a ‘good death’—deciding how to die (e.g., whether to have CPR or not), where to die (e.g., hospital, hospice, elderly, or home—while it is an unlikely option), and who to share the final journey with (e.g., family members). Having an awareness of advance care planning is a good start to having a ‘good death’. Given that advance care planning is a new concept that was only recently introduced in Hong Kong and that most of the previous studies in Hong Kong were conducted in hospital or elderly home settings, it is necessary to conduct a study asking for community-dwelling older adults’ views on advance care planning. While not all the older people in the community are patients, results from community settings can provide a different angle to results collected from a nursing home or hospital setting. By asking older adults’ views on advance care planning in the community, we can better understand what medical concerns affect advance care planning, how to promote advance care planning, and what the older adults are concerned about when they decide whether to use advance care planning. The purpose of this study was to investigate the factors that influence the advance care planning opinions of community-dwelling older adults. Three research questions were formed:

1. What are the demographic factors that influence the preference of using advance care planning?
2. What are the important medical treatment factors that influence the preference of using advance care planning?
3. What are the important decision-making factors that influence the preference of using advance care planning?

## 2. Materials and Methods

### 2.1. Selection of Area

Kwun Tong town center was purposely selected to carry out this study as Kwun Tong has the most extensive aging population in Hong Kong. Furthermore, Kwun Tong is one of the post-war new towns developed in the 1970s under the governorship of Murray MacLehose [24]. According to the latest census in 2016, 210,145 older people were aged 55 or above in Kwun Tong. In the residential area selected for this study, 8501 people were aged 55 or above [3].

### 2.2. Sampling

The targeted respondents were Chinese people living in the Kwun Tong town center aged 55 or above. With the assistance of town councilors, 2446 households were randomly selected within the sampling frame. All of the households with someone aged 55 or above were counted as targeted households and the inhabitant was asked if they would like to be interviewed. If there were no responses at the first visit, two more visits at different times and dates would be arranged; if there was still no answer, the household would be listed as a non-contact. If there was more than one eligible older adult in one household, the ‘next birthday method’ was used to sample one respondent. Of the 2446 households, 1089 households were able to be contacted after three attempts (44.5%). Of the 1089 households who were able to be contacted, 683 were identified as having eligible residents (62.7%), and 282 of the 683 were willing to participate, yielding a response rate 41.3% for the eligible households in this research.

### 2.3. Measurements

Basic demographic information was obtained from each respondent, including age, religious belief, and place of birth. They were also asked about their basic health status (illness and disability). This study did not ask for either personal or household income due to privacy concerns. Then, respondents were asked whether they would use advance care planning, whether they would prepare for death, and whether they had received any death education. An explanation of advance care planning was provided in case the respondents were unaware of it. Four statements related to medical treatments (1–4) and four statements on decision-making (5–8) were also given:

1. I will insist on receiving treatment even it is painful
2. I will terminate treatment to maintain life quality
3. I want to use drugs and other means to alleviate physical pain
4. I want to receive mental support
5. I want to discuss my situation with the medical staff
6. I want to discuss my death decision with my family beforehand
7. If the decision made by my family contradicts my wishes, I will insist on my wishes
8. If I cannot decide, I will trust family rather than the doctor

All questions were dichotomous questions (yes/no) in order to make the questions more manageable for older adults and to avoid triggering any negative feelings about death from the respondents. Since death is a taboo subject in Hong Kong’s society, this study avoided asking too many specific questions so as not to trigger any bad feelings from the respondents. These questions were carefully selected according to different literature. A pilot test was also conducted on 20 older people to ensure the questions were easy to understand.

### 2.4. Analysis

This was a cross-sectional descriptive study. The data was then analyzed using statistical software SPSS 25. Demographic data were combined for ease of analysis. For example, health status was recoded into yes and no. Hong Kong has freedom of religion; different religions exist in Hong Kong. If we break them down, the data will not be enough for analysis. Therefore, it was decided to recode them as ‘has religious beliefs’ and ‘no religious beliefs’. The education level was also recoded as ‘received secondary or above’ and ‘did not receive secondary or above’. Using secondary school as the boundary was based on work from Pang et al., who found that people who did not receive secondary education were less likely to use advance care planning [16]. Pang et al. also found that, because of the previous education system implemented in China and Hong Kong, there was only a small number of tertiary education recipients, the compulsory education in Hong Kong in the 1960s was only six years, and many people could not attend secondary school [24]. Not all of the respondents answered all the questions. Missing data would not be replaced or edited as all variables were nominal data after recording. Basic descriptive statistics, including the mean and standard deviation, were then obtained by using SPSS. To examine the different factors influencing advance care planning use, the Chi-square test was used for independent samples. After that, a multivariable logistic regression was used to find out which characteristics remain significant after controlling for covariates. All characteristics with significant *p* value in the chi-square test were included as a full model. The enter method would be used. Then, non-significant variables would be removed from the model one by one, starting from the highest *p* value, until all variables remained significant. The final model would be reported. The Hosmer–Lemeshow goodness of fit test was used to check the model fit. If the *p* value of the Hosmer–Lemeshow goodness of fit test is smaller than 0.05, the model should be rejected. While stepwise regression is a popular data-mining tool that uses statistical significance to select the explanatory variables to be used in a multiple-regression model, the stepwise method would not be used in this study because the fundamental problem with stepwise regression is that some real explanatory variables that have causal effects on the dependent variable may happen to not be statistically significant, whereas nuisance variables may be coincidentally significant [25]. Instead of using software to eliminate data, using substantive knowledge to guide variable selection is more important and reliable [25].

### 2.5. Ethics

All participants gave their informed consent for inclusion before they participated in the study. The research was approved by the Research Ethics Committee of the authors’ institution and was conducted according to the guidelines of the Declaration of Helsinki.

## 3. Results

### 3.1. Basic Information about the Respondents

A total of 138 males and 144 females were enrolled. The ethnicity of all respondents was Chinese. Their mean age was 72.7 years old and their ages ranged from 55 to 93 years old. A total of 156 respondents had a long-term illness and 26 were disabled. In this study, 55.3% of the respondents had a religious belief; only 21.2% of respondents were born in Hong Kong. Only 13.5% of respondents had heard of advance care planning before the survey (Table 1). For those that had heard of advance care planning before the survey, 63.2% of them had discovered it through the Internet. Among all respondents, only 48 respondents (17%) would consider setting up an advance care planning, after it had been explained to them (Table 1). This study found that while 80 respondents (28.4%) had prepared (or were ready to prepare) for death, only 20 respondents (7.1%) had attended a life and death course or talk (Table 1).

### 3.2. Demographic Characteristics and Advance Care Planning

The study showed no association between advance care planning and gender (*p* = 0.45). The study showed no association between advance care planning and religious belief (*p* = 0.37). For education level, the study showed no association with advance care planning (*p* = 0.08). With an aging population in Hong Kong, people generally have a longer life expectancy. This study divided respondents into two groups according to age: ‘55–69′ and ‘70 or above’. The reason for using 70 as a cut-off was because the mean age in this study was 72.7. There was a significant correlation between the age and advance care planning (*p* = 0.002), suggesting that people in the younger group had a higher chance of using advance care planning than those in the older group (26.9% vs. 12.2%). Using an independent sample *t*-test also obtained a similar result, as the mean age of advance care planning favorers was 69.8 (SD = 8.97), and 73.4 (SD = 7.98) for advance care planning non-favorers (*p* = 0.006). In terms of place of birth, the study showed a significant correlation between the place of birth and advance care planning (*p* = 0.002), suggesting people born in Hong Kong were more likely to use an advance care planning (Table 2).

### 3.3. Health Status and Advance Care Planning

While the study showed no association between advance care planning and disability (*p* = 0.97), there was a significant correlation between long-term illness and advance care planning (*p* = 0.037), suggesting that almost 86.5% of respondents with long-term illness had no plan to use advance care planning (Table 2).

### 3.4. Life and Death Education and Advance Care Planning

Death education is not popular in Hong Kong as death is sensitive subject to talk about. In this study, only 20 respondents had attended a life and death course. However, this study reported that there was a significant correlation between life and death courses and advance care planning (*p* = 0.008), suggesting that people who had received life and death education were more likely to report a preference for using an advance care planning than those who had not attended life and death course or talk (40.0% vs. 16.3%). The study also reported a significant correlation between death preparation and advance care planning (*p* = 0.003) (Table 2).

### 3.5. Medical Treatment, Decision Making and Advance Care Planning

In order to understand how to promote advance care planning in Hong Kong, this study also looked at what medical considerations are associated with advance care planning (Table 3). In terms of treatment, the study found that those who wanted to use advance care planning in the future would have less desire to receive treatment if it would be painful (*p* = 0.009). The reason needs to be studied in the future as the study found that terminating treatment to maintain life quality was not associated with the use of advance care planning (*p* = 0.66). There was also no significant correlation between ‘use of drugs and other means to alleviate physical pain’ and advance care planning (*p* = 0.15). The study reported a significant correlation between mental support and advance care planning (*p* = 0.035). For decision making, the study reported that there was no significant correlation between ‘discussing the situation with medical staff’ and advance care planning (*p* = 0.10). However, the study found that respondents who would like to use advance care planning would be more likely to discuss their death decision with their family beforehand (23.0% vs. 9.1%, *p* = 0.015). Respondents who prefer advance care planning would also be more likely to insist on having wills (25.0% vs. 4.5%, *p* = 0.003). The role of doctors was also important as the study reported a significant correlation between ‘trusting a family rather than the doctor’ and advance care planning (*p* = 0.002).

### 3.6. Multivariable Logistic Regression

Multivariable logistic regression was used to understand which characteristics remain significant after controlling for covariates. All characteristics with a significant *p* value in the chi-square test (Table 2 and Table 3) were included as a full model. The enter method was used. Then, non-significant variables would be removed from the model one by one, starting from the highest *p* value, until all variables remained significant. One of the reasons behind the unfit full model was the small sample size of advance care planning favorers (*n* = 44). The sample is not large enough to include 10 variables in the model simultaneously. Furthermore, gradually eliminating variables from the regression model would make it possible to find a reduced model that best explains the data. The final model reported that five out of 10 variables that were significant in the chi square test remained significant in the regression test. They were ‘illness’, ‘prepared for death’, ‘want to receive mental support’, ‘if the decision contradicts mine, I will insist on your own’ and if I cannot decide, I will trust family rather than the doctor’. This model was fit as the Hosmer–Lemeshow test was not significant (*p* = 0.18), the model therefore should be accepted.

## 4. Discussion

This study was one of the first to ask questions about the advance care planning preferences of community-dwelling older adults in Hong Kong, using a residential sampling method. While only dichotomous questions were asked to avoid triggering any negative feelings about death from the respondents—a taboo and ominous topic in the Chinese cultural context. The results provided valuable insights to help promote advance care planning in Hong Kong.

This study showed that only 17% of the respondents would like to use advance care planning, even after being given an explanation of the meaning of advance care planning. Previous studies conducted by Luk et al. and Pang et al. reported that 88% and 76% of the respondents were in favor of having an advance care planning respectively [15,16], the result of this study was far lower. Community-dwelling older people may not have considered end-of-life arrangements yet due to their age and health. A telephone survey study by Chung et al. also suggested this point, as their study found that only 14.3% of the sample (*n* = 153) had heard of advance care planning before the interviews, and just 60.9% of the sample (*n* = 650) expressed that they would be willing to make an advance care plan after being given an explanation of what advance care planning was during the interviews [11]. The study by Chung et al. was slightly different from this study because the sample of their study was made up of people that were over 30 years old. However, the results of their study were still lower than the data obtained in hospitals or nursing homes.

In terms of age, this study found that a greater percentage of the younger people in the sample (55–69 years old) would like to use advance care planning (*p* = 0.002). Older adults without chronic illnesses (such as high blood pressure and diabetes) were also more likely to use advance care planning (*p* = 0.037). The result from the multivariable logistic regression (Table 4) also reported the same, as it found that the likelihood of using advance care planning in the ‘without illness group’ was 28% lower than in the ‘illness group’ (*p* = 0.001). The results of this study echoed the study by Pang et al. [16]. Given that the mean age of advance care planning favorers was significantly younger than advance care planning non-favorers, it is natural to think that the younger generation without illness or disability is likely to choose advance care planning in order to have their own will realized. In the sample of this study, 66.7% of respondents aged 70 or older had long-term illnesses, which was significantly higher than the group of 55–69-year-olds (33.3%, *p* = 0.030). If we exclude the group of 55–69 year-olds and only analyze the group of people that are 70 years old or older, the data indicate that only 6.3% of advance care planning favorers had long-term illnesses; for advance care planning non-favorers, up to 93.8% had long-term illnesses (*p* = 0.006).

The results of this study are very different from previous studies. Previous studies have shown that older adults strongly favor advance care planning, but these studies were conducted in hospitals or nursing homes, and many of the interviewees were dying patients. For instance, the studies of Luk et al. and Pang et al. were conducted in the hospital and nursing home settings; this might be the reason for the completely different results [15,16]. Older adults receiving treatment in hospitals or nursing homes may be advised on or informed about advance care planning by medical professionals. Older adults facing terminal illnesses must also consider end-of-life arrangements, and they might take advance care planning as an option based on the advice of medical professionals. Therefore, the results obtained in hospitals and nursing homes will differ from the research done in a residential setting.

While the results of this study are distinct from previous studies conducted in non-community-dwelling settings, the results reflect two factors that affect the understanding and awareness of advance care planning. The first is the promotion of death education, and the second is the openness and information acceptance of the respondents. The results of this study implied that death education should be promoted in Hong Kong, as the results suggested that most older adults living in non-hospital or nursing home settings were not ready for death or had never attended any life and death education. A Chinese saying goes, ‘death and life have their determined appointments; riches and honors depend upon heaven’. Older adults with chronic illnesses may feel that life and death are determined by destiny, so they might not pay much attention to death preparation.

Since it is taboo to talk about death publicly in Chinese society, if there is a lack of promotion or public education by medical professionals, the community-dwelling older adults may naturally not understand advance care planning or even consider using it. This is one of the reasons why older-aged respondents with chronic illnesses surprisingly tended to be advance care planning non-favorers in this research, compared to previous studies that were conducted in hospitals or nursing homes, where patients would have chances to receive information about advance care planning. Therefore, this study recommends that more death education should be promoted, so that people will have the appropriate knowledge and emotional preparedness to face their illnesses, as well as illnesses of family members and friends that may be expected or unexpected. The results also indicated that death education could promote advance care planning, as the results showed that the percentage of willingness to use advance care planning in those who had attended a life and death course or talk was higher than the willingness of those that had not (40.0% vs. 16.3%). Those who had prepared for death were also more willing to use advance care planning (30.0% vs. 14.0%). The result from the multivariable logistic regression (Table 4) also reported that the odds of using advance care planning in the ‘prepared for death’ group was 4.94 times higher than those were not (<0.001).

For the openness and information acceptance of the respondents, young and healthy older adults may have more time to and channels through which they can receive information, such as newspapers and the Internet. Given that public discussion of death is taboo in Hong Kong, and advance care planning and advance directives, as the introduction of this article points out, are not separately enacted as a law in Hong Kong, patients need to use advance care planning and advance directives through other common law frameworks [11]. Therefore, the government rarely promotes advance care planning in public broadcasts, such as television and radio. At present, most discussions on advance care planning rely on the public promotion carried out by patient organizations and social welfare agencies or presentations given to dying patients in medical institutions by medical staff, such as hospitals. For community-dwelling older people, this study asked the advance care planning favorers where they first had heard of advance care planning; 63.2% had discovered it through the Internet, 15.8% from traditional press, another 15.8% from medical professionals, and 5.3% from family members and friends (Table 1). As a result, the Internet plays a vital role in spreading this new end-of-life planning concept.

The role of the Internet may explain why the group of those aged 70 years old or above was less willing to use advance care planning. Unlike other countries, the Internet penetration rate of the elderly in Hong Kong is relatively low. According to the Census and Statistics Department, the Internet penetration rate for persons aged between 10 and 54 was close to 100% in 2016, but for persons aged between 55 and 64 years it was 87.7%; for persons aged 65 or above the penetration rate was only 44% [26]. As the Internet is not popular among the elderly, this will reduce their exposure to information. If we compare the study of Chung et al., this conjecture may be true, because the study of Chung et al., which included young people over 30 years old, reported that more people were willing to use advance care planning. Furthermore, since the government has not publicly promoted advance care planning using traditional media, older people seem to have less accessibility to advance care planning information; this might make them advance care planning non-favorers due to a lack of understanding or misunderstanding.

This study also found that the difference in birthplace also has a great effect on the results. For the group who would use advance care planning, the proportion of Hong Kong-born was higher than the group that would not use advance care planning. This is probably related to Hong Kong’s social and political history. After the Second World War, a civil war broke out in China; many mainland Chinese inhabitants fled to Hong Kong. Many of them gave birth to the next generation in Hong Kong. This study found that the place of birth and age were associated at the significant level (*p* = 0.001), with only 26.7% of the group of those aged 70 years old or older having been born in Hong Kong. For the group aged 55 to 69, 73.3% were born in Hong Kong. As Hong Kong was a British colony at that time, the education system was the British system, and most of the schools were run by either Anglican or Catholic churches. These baby boomers were born in Hong Kong and received education locally, they were therefore relatively open-minded, and many of them even accepted the Christian faith. In other words, with the influence of Western education and civilization, the baby boomers in Hong Kong not only have more channels through which to learn about health information (such as the skills of obtaining information by themselves as they are likely to use the Internet) but also have less Chinese cultural identity than the pre-war generation [26,27,28]. Previous studies have shown that people born in Hong Kong generally have a more Western and open way of thinking than people from China [28,29,30]. It may be possible that they are less likely to treat death as taboo and more likely to accept advance care planning. Moreover, the previous studies found that social-economic backgrounds such as sex, religion, or education level are not related to the use of advance care planning in Hong Kong [11,15]. Therefore, it is suggested that future research should focus on the relationship between advance care planning and social status or cultural backgrounds.

Apart from age, birthplace, and life and death education, the results also found that medical care and social support were related to the use of advance care planning. The results suggested that the medical care and social support for end-of-life patients should be improved, as when the respondents were asked the medical questions which related to advance care planning, 27.5% of the respondents who were willing to make advance care planning expressed that they would not want to receive treatment if the treatment would be painful. However, the exact reason for this needs to be studied in the future as this study reported that neither ‘terminate treatment to maintain life quality’ or ‘use drugs and other means to alleviate physical pain’ results were statistically significant. The logistic regression also reported that the results for ‘insist on receiving treatment even it is painful’ were not significant after controlling for covariates.

The study also reported that 20.2% of the respondents willing to make advance care planning expressed that they would like to receive mental support, which was higher than those who said no to advance care planning (5.6%). The result from the multivariable logistic regression (Table 4) also reported that mental support was important in explaining the use of advance care planning as it remained significant after controlling for covariates. Those who would like to receive mental support were 8.1 times more likely to use advance care planning than those who would not want to receive mental support (*p* = 0.011). Based on the results, we can see that medical care and social support for end-of-life patients are important. Therefore, the government should consider improving the medical care and support services for patients. Without them, there could be difficulties in achieving some of the patients’ expressed preferences and wishes [31].

The current study also reported that respondents willing to make an advance care plan were more likely to want to discuss their death decision with family in advance. However, the results also showed that 25% of the advance care planning favorers would insist on their own death decision if the decision contradicted with the wish of their family members. Furthermore, 28.8% of the advance care planning favorers said they would trust their doctor rather than family members if the respondents could not make a decision, regardless of whether this was due to mental or physical health reasons or just disagreements with family members. The percentage was higher than the percentage of people that would trust their family members (12.1%). The multivariable logistic regression also found that these two were the most important for the respondents, as the results reported that the group of people that would insist on their decision being carried out rather than the decision of the family members was 5.33 times more likely to use advance care planning than the group that would trust their family members’ decision (*p* =0.032). The results also reported that the group that would trust the doctor’s decision was 75% less likely to use advance care planning than the group that would trust their family members’ decision (*p* = < 0.001).

The regression results imply two important issues. Firstly, older adults’ empowerment is a critical part of the decision to use advance care planning. The government and medical professionals should think about how to promote patients’ rights in the future. Secondly, this study showed the importance of healthcare professionals providing advice to patients and their family members, especially when the patients are more likely to insist on their own will being carried out when using advance care planning. While the government and the hospital authority should continue to promote advance care planning to patients and in the public community; there should be more education among healthcare professionals regarding advance care planning so as to improve their knowledge and the communication skills required to handle such issues. This is very important as healthcare professionals are on the frontline in terms of informing the public of their medical options [32]. They also play a crucial role in promoting advance care planning, so more education among healthcare professionals is needed.

## 5. Limitations and Future Research

This study has several limitations. While the sample of this research was collected in a residential area in Hong Kong with a random sampling method, this study was a cross-sectional design and was exploratory in nature (only simple dichotomous questions were asked due to feasibility), thus limiting the ability to identify substantial implications. Causality can only be inferred cautiously. Furthermore, the possibility of type 1 error could not be excluded in this study due to the low frequency of advance care planning favorers. This study has tried to recruit the elderly as much as possible. However, because the interviewees were recruited through sampling, it was impossible to know how many people had heard of advance care before the interview. This was different from snowball sampling. Although very few of the interviewees had heard of an advance care plan yet said they would make one, this does not mean that this research is worthless. This research not only reflects the attitude of the interviewees towards advance care planning, but also proves, as Chung et al. found, that the public awareness of advance care planning is low [11].

It should also be noted that due to the legal issue related to the Privacy Ordinance of Hong Kong, it is difficult to obtain all the names and personal details of all residential addresses to generate the sampling frame of all residents in Hong Kong. In other words, it was not possible to do a random sample study of all of Hong Kong [15]. As a result, the location of this research is selected purposely, because Kwun Tong is a district with the largest elderly population in Hong Kong [2,3], and there were open-minded local councilors willing to help and provide the list of all residential addresses in their wards to generate a sampling frame. Although this research was only conducted in one district in Hong Kong, this study was an attempt to survey the unknown waters of the use of advance care planning of the community-dwelling older adults in Hong Kong. When reading this article, readers should also refer to previous studies carried out in other settings in Hong Kong.

For future research, researchers should consider increasing the number of samples when studying advance care planning in a community setting. This can reduce the probability of type 1 error. In addition, Hong Kong’s residents’ awareness of and intention to use advance care planning are low comparing with previous studies in Western countries. It is generally believed that education level and religious belief will affect the results of this type of research, because previous studies found that the favorer rate of advance care planning in the United Kingdom and the United States is higher than that of Hong Kong [33,34]. Whereas Poland and Italy have very low rates and are both have Catholicism as the main religion (70% of the population in Italy is Catholic, while 87% of Poland is Catholic) [34,35,36]. However, in Hong Kong, this study and some previous quantitative studies found that education level and religious beliefs had no significant relationship with preparations for death or advance care planning [11,15]. This may be related to the higher education level of Hong Kong’s residents and Hong Kong’s freedom of religion, as there is no religion playing a dominant role in Hong Kong.

In addition to education and religious beliefs, future research can also focus on the cultural aspect (such as traditional Chinese folklore custom) to study whether culture influences the preparation for death and the use of advance care planning, as previous and current research in Hong Kong reported that education and religious beliefs were not associated with advance care planning. Furthermore, as most previous studies were conducted by medical professionals, they rarely involved cultural issues. Yet, culture is often an important indicator of understanding the public’s acceptance of the policy from a policy-making perspective.

## Figures and Tables

**Table 1:** Characteristics of the study population (N = 282).

		N	%
Gender			
Male		138	48.94
Female		144	51.06
Age			
55–69		110	39.01
70 or above		172	60.99
Illness			
Yes		156	55.32
No		126	44.68
Disability			
Yes		26	9.22
No		256	90.78
Religion			
Yes		156	55.32
No		126	44.68
Education level: Secondary or above			
Yes		74	26.24
No		208	73.76
Born in Hong Kong			
Yes		60	21.28
No		222	78.72
Had heard of advance care planning before the survey			
Yes		38	13.47
No		244	86.53
Where they had first heard of advance care planning (n = 38)			
Family members and friends		2	5.26
Medical professionals		6	15.79
The Internet		24	63.16
Traditional press		6	15.79
Advance care planning preference after explanation			
Yes		48	17.02
No		220	78.01
Missing		14	4.97
Have ever prepared for death			
Yes		80	28.37
No		172	60.99
Missing		30	10.64
Have attended life and death course/talk			
Yes		20	7.09
No		246	87.24
Missing		16	5.67

**Table 2:** Background characteristics of participants by advance care planning.

	Yes (%)	No (%)	*p*
Gender (N = 270)			
Male	26 (19.70)	106 (80.30)	0.45
Female	22 (16.18)	114 (83.82)	
Age (N = 270)			
55–69	28 (26.92)	76 (73.08)	**0.002**
70 or above	20 (12.20)	144 (87.80)	
Illness (N = 270)			
Yes	20 (13.51)	128 (86.49)	**0.037**
No	28 (23.33)	92 (76.67)	
Disability (N = 268)			
Yes	4 (18.18)	18 (81.82)	0.97
No	44 (17.89)	202 (82.11)	
Religion (N = 268)			
Yes	30 (19.74)	122 (80.26)	0.37
No	18 (15.52)	98 (84.48)	
Secondary or above (N = 268)			
Yes	8 (11.11)	64 (88.89)	0.08
No	40 (20.41)	156 (79.59)	
Born in Hong Kong (N = 268)			
Yes	18 (32.14)	38 (67.86)	**0.002**
No	30 (14.15)	182 (85.85)	
Have ever prepared for death (N = 252)			
Yes	24 (30.00)	56 (70.00)	**0.003**
No	24 (13.95)	148 (86.05)	
Have attended life and death course or talk (N = 266)			
Yes	8 (40.00)	12 (60.00)	**0.008**
No	40 (16.26)	206 (83.74)	

**Table 3:** Participants’ medical treatment and decision making according to advance care planning.

	Yes (%)	No (%)	*p*
Will insist on receiving treatment even it is painful (N = 240)			
Yes	22 (13.75)	138 (86.25)	**0.009**
No	22 (27.50)	58 (72.50)	
Will terminate treatment to maintain life quality (N = 222)			
Yes	24 (20.00)	96 (80.00)	0.66
No	18 (17.65)	84 (82.35)	
Use drugs and other means to alleviate physical pain (N = 228)			
Yes	38 (18.81)	164 (81.19)	0.15
No	8 (30.77)	18 (69.23)	
Want to receive mental support (N = 234)			
Yes	40 (20.20)	158 (79.80)	**0.035**
No	2 (5.56)	34 (94.44)	
Want to discuss my situation with medical staff (N = 238)			
Yes	42 (20.00)	168 (80.00)	0.10
No	2 (7.14)	26 (92.86)	
Want to discuss my decision with family beforehand (N = 240)			
Yes	40 (22.99)	134 (77.01)	**0.015**
No	6 (9.09)	60 (90.91)	
If family’s decision contradicts mine, I will insist on my own (N = 212)			
Yes	42 (25.00)	126 (75.00)	**0.003**
No	2 (4.55)	42 (95.45)	
If I cannot decide, I will trust family rather than a doctor (N = 220)			
Yes	14 (12.07)	102 (87.93)	**0.002**
No	30 (28.85)	74 (71.15)	

**Table 4:** Multivariable logistic regression.

		95% CI for OR		
	OR	Lower	Upper	*p*
Illness	0.72	0.33	1.56	**0.001**
Prepared for death	4.94	2.50	9.76	**<0.001**
Want to receive mental support	8.10	1.61	40.61	**0.011**
If the decision contradicts mine, I will insist on my own decision	5.33	1.16	24.64	**0.032**
If I cannot decide, I will trust family rather than the doctor	0.25	0.11	0.54	**<0.001**

## Data Availability

The data are not publicly available due to privacy and ethics approval approved by the Institutional Review Board.
